# Chemical, Biological and Biomedical Aspects of Bioantioxidants

**DOI:** 10.3390/biomedicines11051377

**Published:** 2023-05-06

**Authors:** Sarmistha Saha, Luciano Saso, Aleksei V. Trofimov, Olga I. Yablonskaya

**Affiliations:** 1 Department of Biotechnology, Institute of Applied Sciences & Humanities, GLA University, Mathura 281406, Uttar Pradesh, India; sarmistha.saha@gla.ac.in; 2 Department of Physiology and Pharmacology “Vittorio Erspamer”, Sapienza University of Rome, 00185 Rome, Italy; 3 Emanuel Institute of Biochemical Physics, Russian Academy of Sciences, Ul. Kosygina 4, 119334 Moscow, Russia; olga.yablonsky@gmail.com; 4 Moscow Institute of Physics and Technology, National Research University, Institutskii per. 9, 141701 Moscow, Russia

Bioantioxidants are biologically important antioxidants, a heterogeneous variety of molecules, which are difficult to classify using commonly shared structural features [1,2,3]. Diverse classifications of bioantioxidants consider their origin (synthetic or natural), nature (enzymatic or non-enzymatic), physicochemical properties (lipophilic or hydrophilic), chemical structure (phenolic, polyphenolic, etc.), mechanism of action (chain-breaking or related to pathways such as NRF2/KEAP1) [1]. In biological and biomedical contexts, the major property of bioantioxidants refers to their ability to reduce all chemical species with high Ox/Red potential, which makes these species destructive to biomolecules and body structures [2,3].

The term “Oxidative stress” defines the imbalance between oxidant production and antioxidant defense, which could lead to a wide range of pathological consequences such as atherosclerosis, cancer, and Alzheimer’s disease [4]. In this context, pathologies could be categorized into two groups: one category includes those pathologies in which oxidative stress is the primary cause, such as atherosclerosis. Another category refers to those pathologies in which oxidative stress is the secondary complication, such as Alzheimer’s disease and hypertension [5]. However, the extent of this pathology depends on the redox signaling mechanism. Oxidative stress involves the chemistry of so-called reactive species emanating from oxygen and nitrogen. Overall, two major mechanisms exist for oxidative-stress-induced pathological consequences.

The first mechanism involves the reactive oxygen species (ROS) and reactive nitrogen species (RNS) generation, such as **^•^**OH (hydroxyl radical), ONOO^‒^ (peroxynitrite), and HOCl (hypochlorous acid, followed by other ROS), which are responsible for the oxidation of biomolecules, particularly membrane lipids, structural proteins, and enzymes, leading to cellular dysfunctions and death [6]. In the framework of such a mechanism, the protective function of antioxidants refers mainly to direct interactions with ROS, most prominently to scavenging free radicals [7,8,9]. In this context, the major mechanistic intricacy derives from the inherent sophistication of the chemical behavior of bioantioxidants [7]. Thus, some antioxidants may exhibit prooxidant activity under certain conditions [7]. Therefore, the interplay between oxidative species and antioxidants is not always straightforward. Besides, it is noteworthy that overloading with exogenous chain-breaking antioxidants (e.g., including antioxidants in environmental pollution [10] and in cigarette smoke [11,12]) may represent a certain challenge for toxicology and ecotoxicology in the elucidation of the detailed mechanisms of toxic effects and understanding the pertinent risk factors for living organisms. Finally, the possible metabolic transformations of bioantioxidant molecules may alter their activity, sometimes even to elevated levels [13].

The second oxidative-stress mechanism involves aberrant redox signaling. This may be illustrated by oxidants such as H_2_O_2_, which can act as second messengers in physiological conditions; however, this causes aberrant redox signaling under oxidative stress [14].

Understanding this species-related cellular damage could provide a rationale for therapeutic strategies for antioxidant defense. Biologically active compounds with antioxidant activity, i.e., bioantioxidants, exhibit a wide range of pharmacological applications [15]. Although multiple antioxidant therapeutic strategies are being explored, the practical applications of therapeutics targeting oxidative stress are limited due to a lack of knowledge of their mechanism of action. Extensive research has focused on the antioxidant enzymes and their regulatory pathways, such as the induction of haem oxygenase 1 (HO1), peroxisome proliferator-activated receptor-γ (PPARγ), superoxide dismutase (SOD), and many others with therapeutic applications [16,17,18]. However, at present, only a few clinical trials are ongoing, including ebselen, a glutathione peroxidase (GPX) mimic in phase II (NCT02603081), GC4419, an SOD mimic in phase I (NCT01921426), and sulforaphane, an activator of the Nrf2 transcription factor, in phase II (NCT01335971). Therefore, it is necessary to elucidate the mechanism of action of potential bioantioxidants for further clinical translation. In this context, Nrf2 (NF-E2-related factor 2) is a Cap’n’collar (CNC) transcription factor of bZIP transcription factors (CNC- bZIP) [19]. Nrf2 and its principal negative regulator, the E3 ligase adaptor Kelch-like ECH-associated protein 1 (Keap1), are interconnected in the regulation of intracellular redox homeostasis and inflammation in different pathologies [19]. Under homeostatic conditions, Keap1 binds with ETGE and DLG motifs in the Neh2 domain of Nrf2, which is responsible for their cytoplasmic localization. Keap1 is a substrate adaptor protein for the Cul3-based E3–ligase complex and is involved in ubiquitination via the ubiquitin–proteasome system [20]. However, the cellular stress induced by ROS, xenobiotics, and other electrophiles prevents Nrf2 from interacting with the ubiquitin-conjugating system due to a conformational modification in the E3–ligase complex [21]. After release, Nrf2 translocates into the nucleus, forming a heterodimer with sMaf protein and leading to ARE binding. Thus, it further regulates the expression of cell protective and antioxidant proteins [20]. This Special Issue, entitled “Bioantioxidants in Health and Disease: Chemical, Biological and Biomedical Aspects”, which includes original research articles and review articles, shares insights on the redox signaling pathways in different diseases. The study by Baburina et al. [21] documented the role of prohibitins in regulating oxidative phosphorylation. Prohibitin forms a complex with mitochondrial membrane proteins and some subunits of the respiratory chain complex. The study reported a decreased concentration of prohibitin protein in the heart mitochondria of rats with heart failure; however, astaxanthin protected the rats from heart failure by increasing the prohibitin protein content. The data presented by Badun et al. [22] identified a tritium labeling technique to label natural compounds, namely peat fulvic acid and oxidized lignin derivative for pharmacokinetic studies, for efficient tissue-distribution studies.

Méndez-Valdés et al. [23] summarized the recent studies relating angiotensin II receptors with ischemia/reperfusion injury. Reperfusion damage occurs due to blood flow restoration in the tissue affected by ischemia. Percutaneous coronary intervention (PCI) triggers ROS generation, leading to different cell death pathways, including apoptosis, necrosis, or ferroptosis [24]. Méndez-Valdés et al. [20] reviewed the AT1R blockers targeting Ang II/AT1R axis with the simultaneous ROS burst. They demonstrate the synergistic therapeutic efficacy of AT1R antagonist drugs with other cardiovascular therapeutics for ischemia. Abelli et al. [25] discussed the use of combined antioxidant multitherapy in sepsis, where it has been suggested that oxidative stress and inflammation play a crucial role. The authors reviewed the role of potent antioxidants such as Vitamin C and E, N-acetylcysteine, and selenium for sepsis therapies.

Finally, Ávila et al. [26] proposed using a synergistic antioxidant therapeutic strategy against male infertility. The authors discussed the role of lipid peroxidation, ROS generation, mitochondrial dysfunction, apoptosis, and DNA damage in the pathophysiology of male infertility and how these pathological changes could be overcome by long-chain ω-3 polyunsaturated fatty acids, resveratrol, and some others via the Nrf2 regulatory pathway.

We consider this Special Issue to bring together a wide range of articles contributing to understanding the designing and fabrication of bio-based antioxidant therapeutic strategies.

## References

[1] Vertuani S., Angusti A., Manfredini S. (2004). The antioxidants and pro-antioxidants network: An overview. Curr. Pharm. Des..

[2] Brainina K., Stozhko N., Vidrevich M. (2019). Antioxidants: Terminology, methods, and future considerations. Antioxidants.

[3] Tirzitis G., Bartosz G. (2010). Determination of antiradical and antioxidant activity: Basic principles and new insights. Acta Biochim. Pol..

[4] Forman H.J., Zhang H. (2021). Targeting oxidative stress in disease: Promise and limitations of antioxidant therapy. Nat. Rev. Drug Discov..

[5] Sies H. (2020). Oxidative stress: Concept and some practical aspects. Antioxidants.

[6] Firuzi O., Miri R., Tavakkoli M., Saso L. (2011). Antioxidant therapy: Current status and future prospects. Curr. Med. Chem..

[7] Fedorova G.F., Kancheva V.D., Menshov V.A., Naumov V.V., Vasil’ev R.F., Veprintsev T.L., Trofimov A.V., Tsaplev Y.B., Yablonskaya O.I. (2016). Exogenous and endogenous mediators of oxygen metabolism: Alternatives for chemical and biological activity. Stud. Nat. Prod. Chem..

[8] Balansky R., La Maestra S., Kancheva V.D., Trofimov A.V., Djongov L., De Flora S. (2021). Clastogenic effects of cigarette smoke and urethane and their modulation by olive oil, curcumin and carotenoids in adult mice and foetuses. Food Chem. Toxicol..

[9] Fedorova G.F., Menshov V.A., Trofimov A.V., Vasil’ev R.F. (2009). Facile chemiluminescence assay for antioxidative properties of vegetable lipids: Fundamentals and illustrative examples. Analyst.

[10] Belyakov V.A., Fedorova G.F., Vasil’ev R.F. (1993). A kinetic chemiluminescence study of the antioxidants evolved from polymeric materials into the gaseous phase. J. Photochem. Photobiol. A Chem..

[11] Fedorova G.F., Menshov V.A., Trofimov A.V., Tsaplev Y.B., Vasil’ev R.F., Yablonskaya O.I. (2017). Chemiluminescence of cigarette smoke: Salient features of the phenomenon. Photochem. Photobiol..

[12] Palmina N.P., Maltseva E.L., Chasovskaya T.E., Kasparov V.V., Bogdanova N.G., Menshov V.A., Trofimov A.V. (2014). Effects of different phases of cigarette smoke on lipid peroxidation and membrane structure in liposomes. Aust. J. Chem..

[13] Menshov V.A., Yablonskaya O.I., Trofimov A.V., Kancheva V.D. (2021). Transformation of the antioxidant properties of tar from tobacco smoke in metabolic processes: Model chemiluminescence study. Russ. J. Phys. Chem. B.

[14] Flohé L. (2020). Looking back at the early stages of redox biology. Antioxidants.

[15] Kancheva V.D., Kasaikina O.T. (2013). Bio-antioxidants—A chemical base of their antioxidant activity and beneficial effect on human health. Curr. Med. Chem..

[16] Krönke G., Kadl A., Ikonomu E., Blüml S., Fürnkranz A., Sarembock I.J., Bochkov V.N., Exner M., Binder B.R., Leitinger N. (2007). Expression of heme oxygenase-1 in human vascular cells is regulated by peroxisome proliferator- activated receptors. Arterioscler. Thromb Vasc. Biol..

[17] Rojo A.I., Salinas M., Martin D., Perona R., Cuadrado A. (2004). Regulation of Cu/Zn- superoxide dismutase expression via the phosphatidylinositol 3 kinase/Akt pathway and nuclear factor- kappaB. J. Neurosci..

[18] Telkoparan-Akillilar P., Suzen S., Saso L. (2019). Pharmacological applications of Nrf2 inhibitors as potential antineoplastic drugs. Int. J. Mol. Sci..

[19] Saha S., Buttari B., Panieri E., Profumo E., Saso L. (2020). An overview of Nrf2 signaling pathway and its role in inflammation. Molecules.

[20] Kansanen E., Kuosmanen S.M., Leinonen H., Levonen A. (2013). The Keap1-Nrf2pathway: Mechanisms of activation and dysregulation in cancer. Redox Biol..

[21] Baburina Y., Krestinin R., Odinokova I., Fadeeva I., Sotnikova L., Krestinina O. (2021). The identification of prohibitin in the rat heart mitochondria in heart failure. Biomedicines.

[22] Badun G.A., Chernysheva M.G., Zhernov Y.V., Poroshina A.S., Smirnov V.V., Pigarev S.E., Mikhnevich T.A., Volkov D.S., Perminova I.V., Fedoros E.I. (2021). A Use of tritium-labeled peat fulvic acids and polyphenolic derivatives for designing pharmacokinetic experiments on mice. Biomedicines.

[23] Méndez-Valdés G., Pérez-Carreño V., Bragato M.C., Hundahl M., Chichiarelli S., Saso L., Rodrigo R. (2023). Cardioprotective mechanisms against reperfusion injury in acute myocardial infarction: Targeting angiotensin II receptors. Biomedicines.

[24] Yellon D., Hausenloy D. (2007). Myocardial Reperfusion Injury. N. Engl. J. Med..

[25] Abelli J., Méndez-Valdés G., Gómez-Hevia F., Bragato M.C., Chichiarelli S., Saso L., Rodrigo R. (2022). Potential antioxidant multitherapy against complications occurring in sepsis. Biomedicines.

[26] Ávila C., Vinay J.I., Arese M., Saso L., Rodrigo R. (2022). Antioxidant intervention against male infertility: Time to design novel strategies. Biomedicines.

